# Gorlin-Goltz syndrome without cutaneous manifestations

**DOI:** 10.11604/pamj.2021.39.239.30886

**Published:** 2021-08-13

**Authors:** Rohan Kumar Singh, Gaurav Vedprakash Mishra

**Affiliations:** 1 Department of Radiodiagnosis, Jawaharlal Nehru Medical College, Datta Meghe Institute of Medical Sciences, Sawangi (Meghe), Wardha, India

**Keywords:** Computerized tomography (CT) scan, Gorlin-Goltz syndrome, odontogenic keratocyst, PTCH gene mutation

## Image in medicine

A 29-year-old male visited the outpatient department of Acharya Vinoba Bhave Rural Hospital, Sawangi with chief complaints of swelling in the left lower jaw for 15 days. He had a past history of similar swelling on the right side of the jaw, for which he underwent intervention. The patient was referred from oral surgery to the Department of Radiodiagnosis for computed tomography scan of head and neck to know the extension of the lesion and underlying bony erosion. There was no significant family history or cutaneous manifestations. On computed tomography, there were multiple cystic lesions in the left hemimandible and maxilla. Histopathology revealed it to be Odontogenic keratocysts. Other findings included bilamellar falx cerebri and tentorium cerebelli calcifications, bridging sella, intracranial lipoma, spina bifida of C6 vertebra. The scalp was thickened with multiple foci of calcifications. Diagnosis of Gorlin-Goltz syndrome was made.

**Figure 1:**
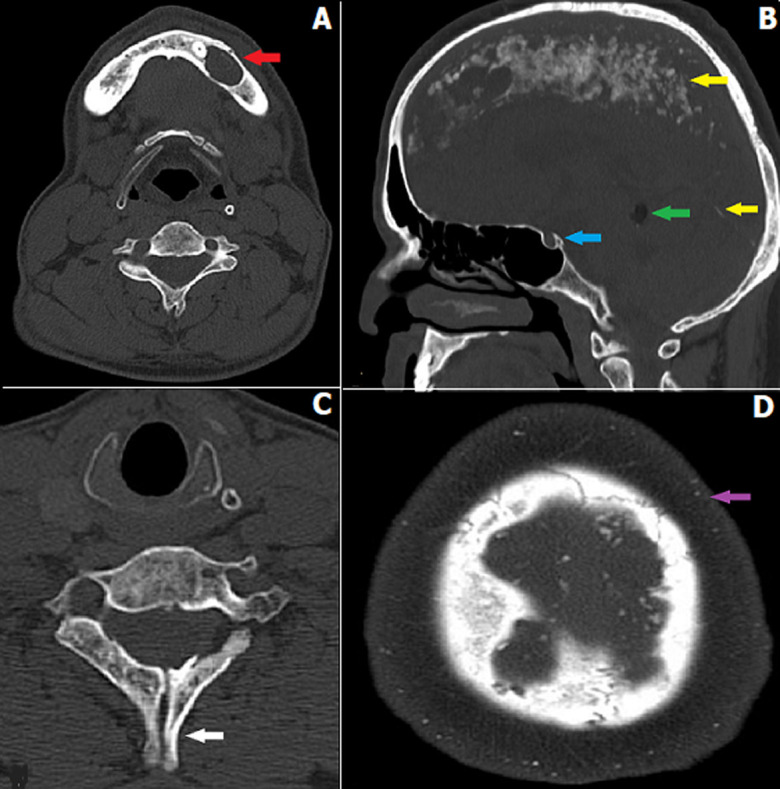
A) computed tomography image showing multiple cystic lesions in the left hemimandible and maxilla consistent with odontogenic keratocysts (red arrow); B) bilamellar falx cerebri and tentorium cerebelli calcifications (yellow arrow), bridging sella (blue arrow), intracranial lipoma (green arrow); C) spina bifida of C6 vertebra (white arrow); D) thickened scalp with multiple calcific foci (purple arrow); diagnosis of Gorlin-Goltz syndrome was made

